# A Study of the Expression of Small Conductance Calcium-Activated Potassium Channels (SK1-3) in Sensory Endings of Muscle Spindles and Lanceolate Endings of Hair Follicles in the Rat

**DOI:** 10.1371/journal.pone.0107073

**Published:** 2014-09-05

**Authors:** Fiona Shenton, Guy S. Bewick, Robert W. Banks

**Affiliations:** 1 School of Biological & Biomedical Sciences, Durham University, Durham, United Kingdom; 2 School of Medical Sciences, University of Aberdeen, Aberdeen, United Kingdom; University of South Florida, United States of America

## Abstract

Processes underlying mechanotransduction and its regulation are poorly understood. Inhibitors of Ca^2+^-activated K^+^ channels cause a dramatic increase in afferent output from stretched muscle spindles. We used immunocytochemistry to test for the presence and location of small conductance Ca^2+^-activated K^+^ channels (SK1-3) in primary endings of muscle spindles and lanceolate endings of hair follicles in the rat. Tissue sections were double immunolabelled with antibodies to one of the SK channel isoforms and to either synaptophysin (SYN, as a marker of synaptic like vesicles (SLV), present in many mechanosensitive endings) or S100 (a Ca^2+^-binding protein present in glial cells). SK channel immunoreactivity was also compared to immunolabelling for the Na^+^ ion channel ASIC2, previously reported in both spindle primary and lanceolate endings. SK1 was not detected in sensory terminals of either muscle spindles or lanceolate endings. SK2 was found in the terminals of both muscle spindles and lanceolate endings, where it colocalised with the SLV marker SYN (spindles and lanceolates) and the satellite glial cell (SGC) marker S100 (lanceolates). SK3 was not detected in muscle spindles; by contrast it was present in hair follicle endings, expressed predominantly in SGCs but perhaps also in the SGC: terminal interface, as judged by colocalisation statistical analysis of SYN and S100 immunoreactivity. The possibility that all three isoforms might be expressed in pre-terminal axons, especially at heminodes, cannot be ruled out. Differential distribution of SK channels is likely to be important in their function of responding to changes in intracellular [Ca^2+^] thereby modulating mechanosensory transduction by regulating the excitability of the sensory terminals. In particular, the presence of SK2 throughout the sensory terminals of both kinds of mechanoreceptor indicates an important role for an outward Ca^2+^-activated K^+^ current in the formation of the receptor potential in both types of ending.

## Introduction

Ca^2+^-activated K^+^ channels (SK and BK channels, collectively K_Ca_) are known to play various roles that involve repolarisation of cell membranes, including the regulation of firing rates in central neurons, of smooth muscle tone, and of synaptic transmission [1]. They have been described in a variety of other cell types, including dorsal-root ganglion cells [2], [3], though there are conflicting reports about the possible occurrence of K_Ca_ channels in sensory terminals of low-threshold mechanoreceptors, in particular those of the mammalian muscle spindle [4], [5]. Our own interest in this possibility arose from our work on the small (50 nm), clear vesicles present in mammalian mechanosensory terminals [6], [7]. Despite wide variation in form, associated accessory cells, and function of the terminals, all of them seem to possess a population of the vesicles [8], indicating the existence of an important common underlying mechanism. The vesicles share many properties with those of presynaptic terminals, but as the sensory terminals are emphatically not synaptic we refer to the vesicles as synaptic-like (SLV). Using sensory endings of rat muscle spindles as a model of the role of SLVs we have presented evidence that they are involved in autogenic modulation of sensory-ending excitability, mediated by glutamate released from SLVs during their recycling [6]. This presynaptic similarity of mechanosensory endings prompted us to investigate Ca-dependent mechanisms that might regulate SLV recycling, and/or afferent firing.

As with the similar vesicles in presynaptic terminals, fusion of SLVs with the sensory terminal membrane is Ca^2+^-dependent, and blocking Ca^2+^ influx with inorganic ions (Co^2+^ or Ni^2+^/Cd^2+^) severely inhibits or abolishes the sensory response in muscle spindles [6]. More specific blocking of P/Q-type channels with ω-agatoxin IVA or ω-conotoxin MVIIC powerfully increased firing rates (2–3 fold approximately) in response to stretch. A similar effect was produced if either BK or SK channels were blocked with charybdotoxin, iberiotoxin or apamin [9], [10].

Here we investigate the expression of SK1-3 in sensory terminals of muscle spindles and in lanceolate endings of hair follicles using immunocytochemistry. The synaptic vesicle protein synaptophysin (SYN) was used as a marker of sensory terminals, which show strong immunoreactivity to SYN presumably because of their SLV content. In addition, as a further marker of the sensory terminals, we also examined the location of immunoreactivity to the candidate mechanotransducer channel component ASIC2, also known as BNaC1. We have previously found immunoreactivity to ASIC2 in the sensory endings of spindles, where it colocalises with that to SYN [11]; and immunoreactivity to the BNaC1α isoform has been reported in cutaneous mechanoreceptor endings, including the lanceolate (or palisade) ending of the hair follicle [12]. In the lanceolate ending, unlike the spindle primary ending, individual terminals are closely invested by satellite glial cells (SGCs) in an association so intimate that the SGCs may usefully be considered to be a non-neural component of the ending. We used immunoreactivity to the Ca^2+^-binding protein S100, often regarded as a marker of glial cells [e.g. 13], to identify SGCs.

SK1 expression has recently been reported in the dorsal-root ganglion cells and sensory receptor cells of the special sense organs in zebrafish (*Danio*), though post-cranial sensory endings were not described [14]. We did not detect SK1 in either muscle-spindle or lanceolate endings, apart from some unidentified axon-like structures, whereas immunoreactivity to SK2 was present in both spindle terminals and lanceolate endings. Immunoreactivity to SK3 was found in the lanceolate endings in a pattern consistent with a predominantly, or exclusively, SGC expression.

## Materials and Methods

### Ethics Statement

All procedures were carried out in accordance with UK legislation: Animals (Scientific Procedures) Act, 1986 and all possible care was taken to ameliorate suffering. The study was carried out under UK Home Office Project Licence no. PPL 60/3963, with the approval of Durham University’s Life Sciences Ethical Review Process Committee, granted 2010.

### Tissue Preparation and Imaging

**Figure 1:**
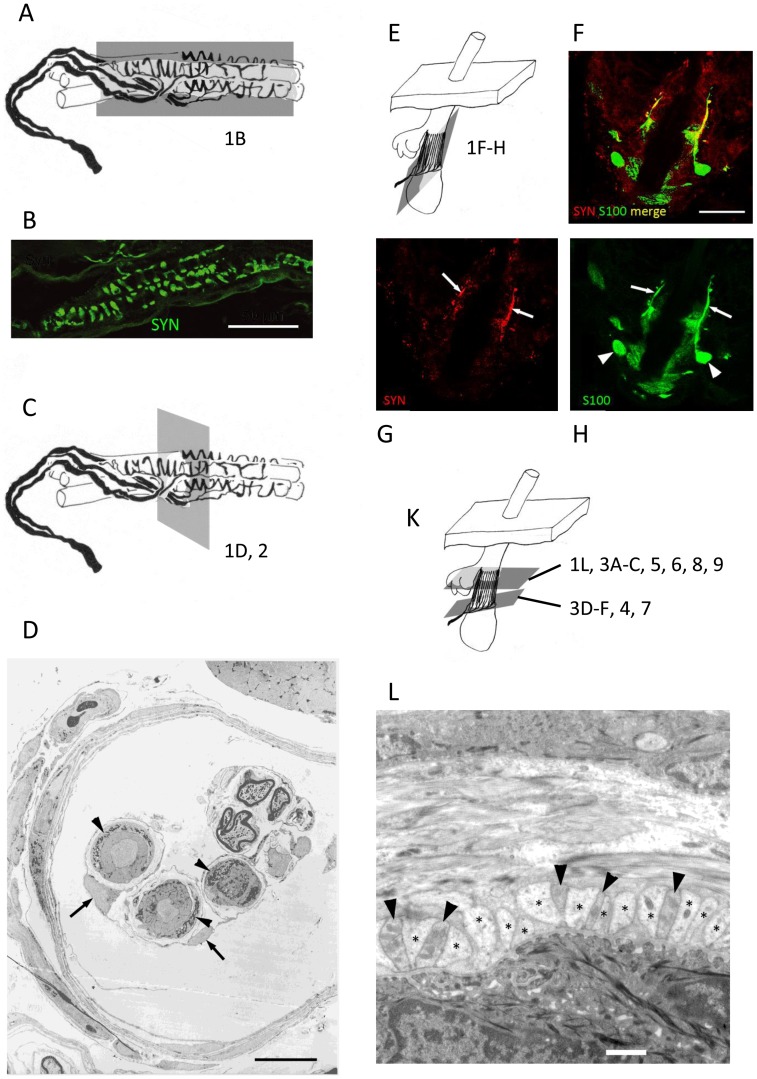
Key structural elements of primary endings in muscle spindles and lanceolate endings on hair follicles. Schematic diagrams of a muscle spindle primary ending, annulo-spiral in form (A, C) and of a lanceolate ending forming a palisade-like structure around a hair follicle (E, K). Representative planes of section of subsequent figures and of the remaining parts of this figure are shown on the diagrams. Immunoreactivity for synaptophysin is apparently restricted to the sensory terminals of both spindle primary (B) and lanceolate (arrows in G) endings. In the muscle spindle, the very large sensory terminals (arrowheads in D) are wound around specialised intrafusal muscle fibres. Surrounding, but not in contact with the sensory ending are inner capsule cells (arrows in D). In the lanceolate ending, the much smaller individual terminals (arrowheads in L) are sandwiched closely between processes of accessory glial cells (asterisks in L). The glial cells, both processes (arrows in H) and cell bodies (arrowheads in H) are immunoreactive for S100. Glial cell processes and lanceolate terminals are so small that in longitudinal section anti-synaptophysin and anti-S100 antibodies can appear to colocalise in a single confocal plane (G and H merged in F). Imaging techniques: B, single plane confocal laser scanning image, scale 50 µm; D, transmission electron micrograph, scale 10 µm; F–H, single plane confocal laser scanning images, scale 20 µm; L, transmission electron micrograph, scale 1 µm.

Adult rats (2) were deeply anaesthetized with sodium pentobarbitone (45 mg kg^−1^, I.P.) and fixed by transcardial perfusion with 4% (w/v) formaldehyde (from paraformaldehyde) in 0.1 M phosphate buffer, pH 7.4. Tissue samples, taken from the pinnae and from a spindle-rich region of the deep masseter muscles of each animal, were cryoprotected by immersion in 30% sucrose in 0.1 M phosphate buffer overnight, before being placed in moulds containing Tissue-Tek and rapidly frozen in isopentane cooled to −160°C with liquid N_2_. 10 µm thick cryosections were cut using a Leica CM 1850 UV cryostat. Sections of skin from the inner surfaces of the pinnae were double labelled with one of four groups of antibody (Ab) combinations: 1) anti-SK channel (1.25–5 µg/ml, goat or rabbit polyclonals, Santa Cruz Biotechnology/Alomone Laboratories) + anti-SYN (1 µg/ml, mouse monoclonal, Millipore); 2) anti-SK + anti-S100 (1∶400, mouse monoclonal, Santa Cruz Biotechnology); 3) anti-ASIC2 (5 µg/ml, goat polyclonal, Santa Cruz Biotechnology) + anti-SYN; 4) anti-ASIC2+ anti-S100. Sections were incubated with primary antibodies for 48 hrs at 4°C. Secondary antibodies were Alexa Fluor (AF) conjugated antibodies (all at 1∶250 dilution, AF 594 donkey anti-goat and AF 488 donkey anti-mouse or AF 594 goat anti-rabbit and AF 488 goat anti-mouse, Invitrogen). Incubations with secondary antibodies were for 1 hr at ambient temperature (circa 20°C). Sections of muscle were incubated only with combinations 1) and 3) as there are no SGCs associated with spindle sensory endings. Channel antibodies pre-incubated with the peptides to which they had been raised were used as controls. Sections were viewed and optically sectioned with a Leica SP5 Confocal Laser Scanning Microscope, using a x63 NA 1.4 objective. Serial confocal planes (Z-stacks) used for movies, animations and reconstructions were 0.5 µm apart. A diagrammatic survey of the key structural components of the primary ending and the lanceolate ending in relation to planes of imaging is given in Figure 1.

### Colocalisation Statistical Analysis

To assess the colocalisation of SK and ASIC2 immunoreactivity with either anti-S100 or anti-SYN labelling, we calculated Manders’ correlation coefficients (M_x_, M_y_) [15], with automatic thresholding, for pairs of images of single confocal planes, using Volocity software (Perkin-Elmer, Cambridge, UK; automatic thresholding is implemented according to the protocol of Costes et al. [16], in Volocity). Regions of interest (ROIs) were drawn freehand to include sensory endings or, where appropriate, SGC bodies and to exclude most of the unlabelled background so that thresholds were not unduly biased by pixels with 0, or close to 0, intensity. In the present data, M_x_ is always the summed intensities in the green channel (AF 488) for pixels above threshold in both channels, as a proportion of the total intensities for all pixels in the green channel; and M_y_ is the equivalent for the red channel (AF 594). To clarify the presentation of the quantitative data in the subsequent results, M_x_ and M_y_ are further specified according to the immunoreactivities being compared, e.g. M_x_ = M_SK2/SYN_ signifies the proportion of total intensities of thresholded pixels immunopositive for SYN that are also immunopositive for SK2, and the complementary M_y_ = M_SYN/SK2_ signifies the proportion of total intensities of thresholded pixels immunopositive for SK2 that are also immunopositive for SYN. Merged images were created using ImageJ (http://imagej.nih.gov/ij National Institutes of Health, Bethesda, MD). Calculation of Manders’ coefficients effectively normalises fluorescent intensities, so reducing variability arising from differences in absolute intensities from ending to ending and preparation to preparation. The coefficients can take values between 0 and 1, and are usually not distributed normally. We therefore made statistical comparisons using Mann-Whitney *U*. Sample sizes (n values) refer to numbers of sensory endings.

### Colocalisation Visualisation and 3D Surface Rendering

Interpretation of the results of immunolabelling in the lanceolate endings of hair follicles is more complicated than in the sensory endings of muscle spindles, due to the smaller size of the lanceolate’s sensory terminals and their close, parallel association with thin processes of SGCs, the distance between the opposed membranes being well below the resolution of the light microscope [17]. Care was needed, therefore, to avoid the possibility of misinterpreting proximity for colocalisation, which is a particular problem with longitudinal sections (Fig. 1F). In order to minimise this problem, data used in the quantitative analysis of Table 1 were taken only from image planes close to transverse with respect to the sensory terminals. Longitudinal and oblique sections were used exclusively for qualitative observations. Qualitative interpretation was further facilitated in more complex sections by viewing Z-stacks of images sequentially in a movie, making it much easier to follow particular structures in 3 dimensions. Some of these were subsequently converted to surface-rendered 3D objects using Imaris software (Imaris 7.7 with colocalisation; Bitplane, Belfast, UK) to investigate spatial relationships between labels further and visualise the colocalisation. Examples of both these treatments are appended as supplementary files. The validity of our interpretation of colocalisation is illustrated by the lack of colocalisation volume when sections were double labelled with anti-SYN (terminals) and anti-S100 (glia), and 3D surface-rendering found no colocalisation volumes present (Movies S1 and S2).

**Table 1:** Colocalisation analysis using Manders’ coefficients for double-labelled immunofluorescence in muscle spindles and lanceolate endings.

muscle spindles																					
channel	Ab	coloc coeff			n																
green	SYN	M_X_ = M_SK2/SYN_	0.74		8																
red	SK2	M_Y_ = M_SYN/SK2_	0.88																		
green	SYN	M_X_ = M_SK3/SYN_	0.01																		
red	SK3	M_Y_ = M_SYN/SK3_	0.47		2																
green	SYN	M_X_ = M_ASIC2/SYN_	0.64																		
red	ASIC2	M_Y_ = M_SYN/ASIC2_	0.78		10																
**lanceolate endings**																					
	**data**														**statistical tests**						
**channel**	**Ab**	**coloc coeff**				**n**			**Ab**		**coloc coeff**		**n**							***U***	***P***
green	SYN	M_X_ = M_SK2/SYN_		0.90					S100		M_X_ = M_SK2/S100_	0.81			M_SK2/SYN_			*v* M_SK2/S100_		61	<0.05
red	SK2	M_Y_ = M_SYN/SK2_		0.81			15		SK2		M_Y_ = M_S100/SK2_	0.96	16		M_SYN/SK2_			*v* M_S100/SK2_		27.5	<0.01
green	SYN	M_X_ = M_SK3/SYN_		0.33					S100		M_X_ = M_SK3/S100_	0.42			M_SK3/SYN_			*v* M_SK3/S100_		63.5	ns
red	SK3	M_Y_ = M_SYN/SK3_		0.63			11		SK3		M_Y_ = M_S100/SK3_	0.88	15		M_SYN/SK3_			*v* M_S100/SK3_		12	<0.01
green	SYN	M_X_ = M_ASIC2/SYN_		0.66					S100		M_X_ = M_ASIC2/S100_	0.48			M_ASIC2/SYN_			*v* M_ASIC2/S100_		98	ns
red	ASIC2	M_Y_ = M_SYN/ASIC2_		0.67			20		ASIC2		M_Y_ = M_S100/ASIC2_	0.79	15		M_SYN/ASIC2_			*v* M_S100/ASIC2_		95	ns

Abbreviations: Ab, antibody; coloc coeff, colocalisation coefficient; M_X_, M_Y_, Manders’ coefficients; SYN, synaptophysin.

## Results

### SK2

We begin with SK2 as we found anti-SK2 immunoreactivity in the terminals both of sensory endings of muscle spindles and lanceolate endings of hair follicles. In addition, anti-SK2 immunoreactivity was present in lanceolate-ending SGCs. Control sections incubated with anti-channel antibodies pretreated with the respective antigenic peptides showed no immunofluorescence.

#### SK2 immunoreactivity in muscle spindles

**Figure 2:**
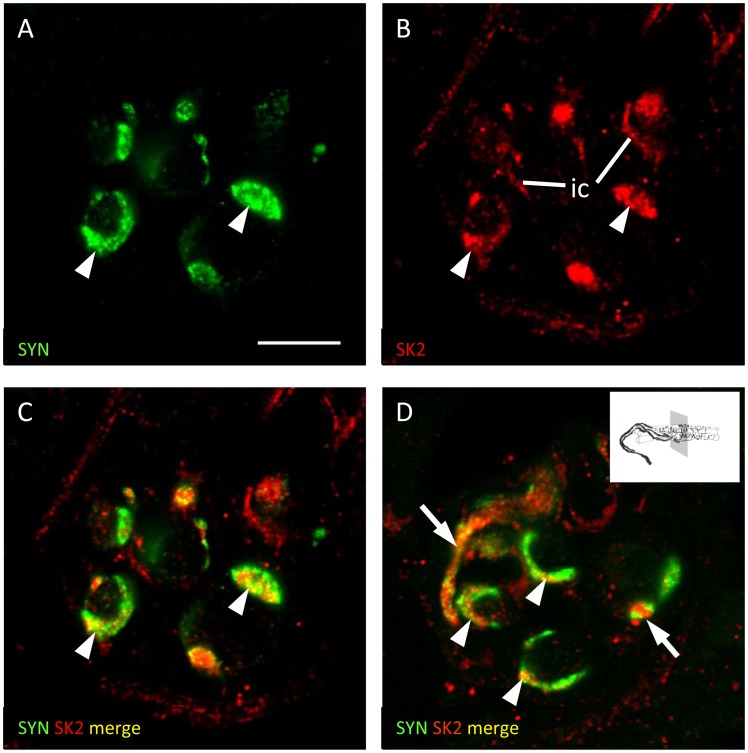
Evidence for the expression of SK2 in muscle spindle sensory endings. Muscle-spindle sensory endings double labelled with anti-synaptophysin (SYN, green channel) and anti-SK2 (red channel) antibodies. In one ending the green and red channels are shown separately (A and B), as well as merged (C). In a second ending only the merged channels are shown (D). Partial colocalisation of SYN and SK2 immunoreactivity is evident in the sensory terminals (arrowheads), and in what are probably unmyelinated preterminal branches of the parent sensory nerve fibre (arrows). SK2 immunoreactivity can also be detected in inner capsule cells (ic). Scale = 10 µm.

SK2 immunoreactivity was detected in sensory terminals and other, less well defined structures, probably inner capsule cells (Fig. 2A–C). Pre-terminal axons are rarely included in 10 µm thick sections of muscle spindles, owing to their very restricted distribution. Nevertheless, in one section, SK2 immunoreactivity was present in what had the structural characteristics of a pre-terminal axon (Fig. 2D). Sensory terminals were identified by their size, shape and positive immunoreactivity for SYN. Manders’ coefficients (SYN, green channel; SK2, red channel) were both high (mean M_x_ = M_SK2/SYN_ = 0.74; mean M_y_ = M_SYN/SK2_ = 0.88, n = 8) for ROIs drawn as single envelopes enclosing all recognisable sensory terminals in each section, indicating strong colocalisation of SK2 and SYN in the terminals. For comparison, we also carried out double labelling with antibodies against SYN (green channel) and ASIC2 (red channel). We have previously described ASIC2 immunoreactivity in the spindle sensory terminals, as a putative component of the mechanotransduction channel [11]. The SYN/SK2 immunoreactivity colocalisation of ROIs enclosing sensory terminals was at least as strong as that for SYN/ASIC2 (mean M_x_ = M_ASIC2/SYN_ = 0.64; mean M_y_ = M_SYN/ASIC2_ = 0.78, n = 10).

#### SK2 immunoreactivity associated with hair follicle sensory innervation

**Figure 3:**
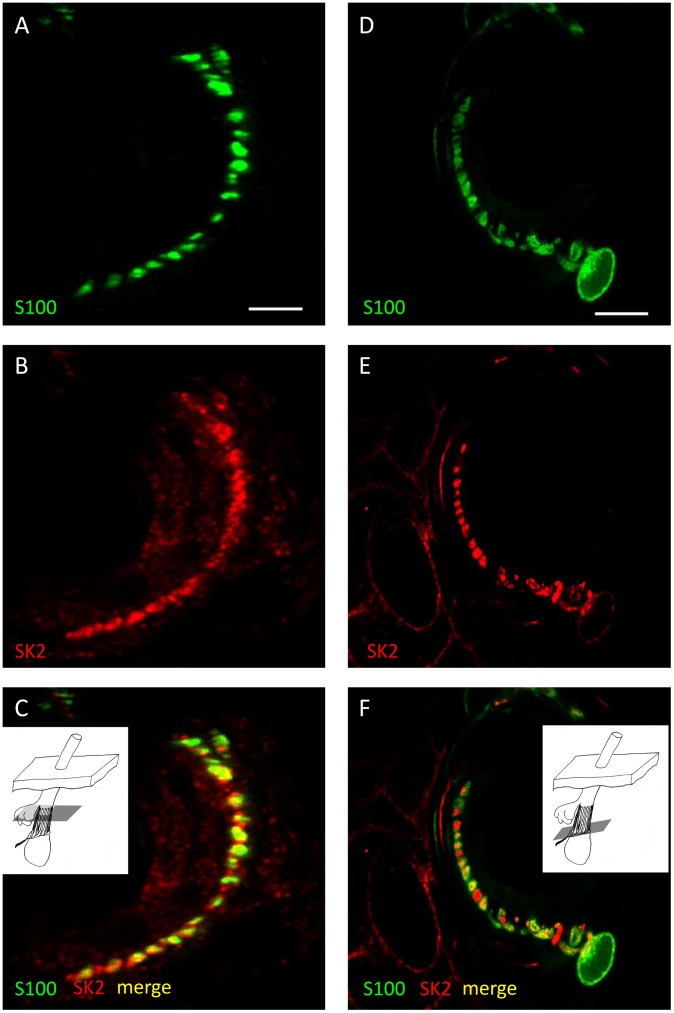
Evidence for the expression of SK2 in sensory terminals and glial cells of lanceolate endings. Lanceolate endings double labelled with anti-synaptophysin (SYN, green channel) and anti-SK2 (red channel) antibodies (A–C) and with anti-S100 (green channel) and anti-SK2 (red channel) antibodies (D–F). In each case the green (A, D) and red (B, E) channels are shown separately, as well as merged (C, F). Partial colocalisation of both SYN and S100 with SK2 immunoreactivity indicates that SK2 is present in sensory terminals and satellite glial cells (SGCs). Scale (in A, D) = 10 µm.

**Figure 4:**
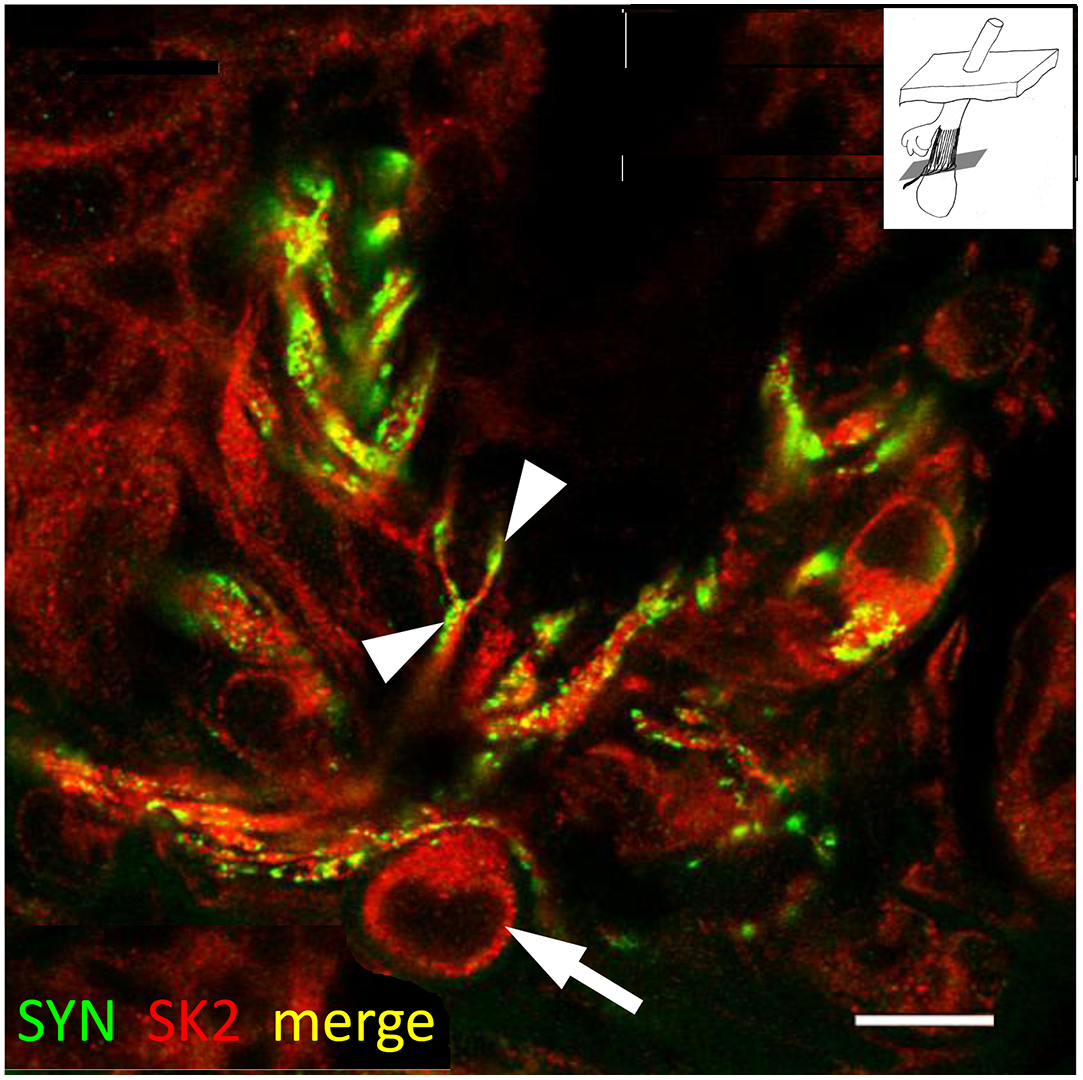
Further evidence for the expression of SK2 in lanceolate sensory terminals and glial cells. An oblique section of a lanceolate ending double labelled with anti-synaptophysin (SYN, green channel) and anti-SK2 (red channel) antibodies in a merged image. Note especially the satellite glial cell (SGC) body labelled only with anti-SK2 antibody (arrow), and the double labelled branched axon and terminals (arrowheads), confirming the expression of SK2 in both sensory terminals and SGCs. Scale = 10 µm.

Examination of pairs of images taken in the red (SK2) and green (SYN or S100) channels, as well as corresponding merged images, showed at least partial colocalisation of SK2 immunoreactivity with both SYN and S100 immunoreactivity in sections passing through the palisade-like ring of sensory terminals and SGC processes of lanceolate endings (Fig. 3). Manders’ coefficients were: for SYN/SK2 (mean M_x_ = M_SK2/SYN_ = 0.90; mean M_y_ = M_SYN/SK2_ = 0.81, n = 15); and for S100/SK2 (mean M_x_ = M_SK2/S100_ = 0.81; mean M_y_ = M_S100/SK2_ = 0.96, n = 16). Corresponding pairs of coefficients differed significantly (M_x_) or highly significantly (M_y_) (Table 1). Collectively, these results indicate that SK2 is expressed in both the sensory terminals and the SGCs. Confirmation was provided by sections passing through the bulb regions of hair follicles, where SGCs and their initial processes could be seen, prior to their association with sensory terminals or axons. Thus, SK2 immunoreactivity was present in the cell bodies (cytoplasm) and processes of SGCs as well as in axons and their terminals, whereas SYN immunoreactivity was present only in the axons and terminals (Fig. 4 and Movies S3,S4).

**Figure 5:**
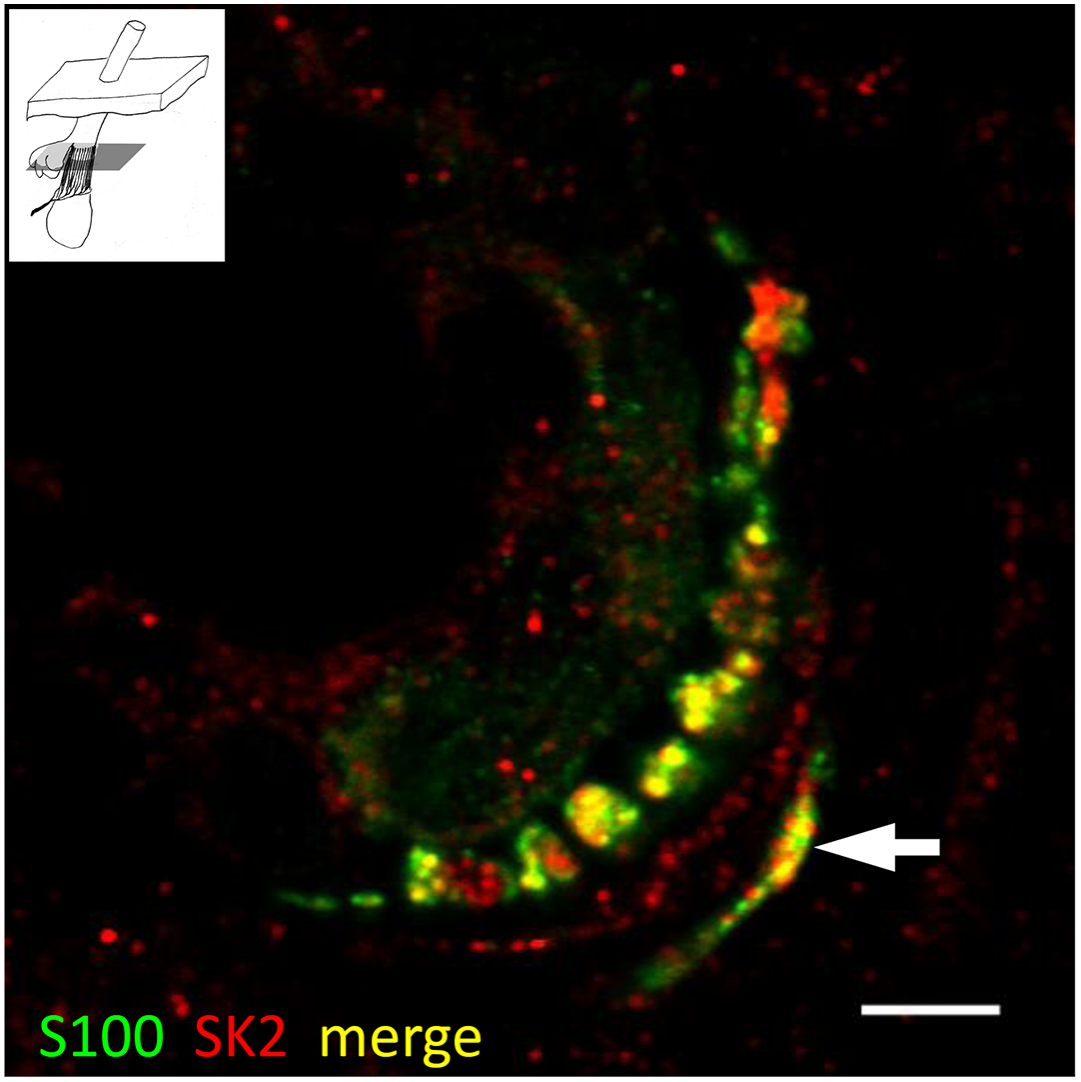
SK2 may be expressed in circumferential as well as lanceolate endings. A lanceolate ending double labelled with anti-S100 (green channel) and anti-SK2 (red channel) antibodies in a merged image. The structure indicated by an arrow may be part of a circumferential ending. Scale = 5 µm.

In addition we sometimes found SK2 immunoreactivity associated with hair follicles, but outside the lanceolate endings themselves (as defined by the sensory terminals and SGCs). In individual sections it was usually not possible to tell whether this was associated with pre-terminal axons of the lanceolate endings, though in some cases the structures involved appeared to be portions of circumferential endings (Fig. 5). SK2 immunoreactivity was also shown by cells of the sebaceous glands and non-neural cells at the base of the follicles, whereas the inner and outer sheath cells did not show reactivity.

### SK3

#### SK3 in muscle spindles

We did not detect immunoreactivity to SK3 in the terminals of muscle spindle sensory endings. However, we cannot rule out the possibility that SK3 is present in the pre-terminal axonal branches, in view of the difficulty of finding them in our sections, as described above.

#### SK3 associated with hair follicle sensory innervation

**Figure 6:**
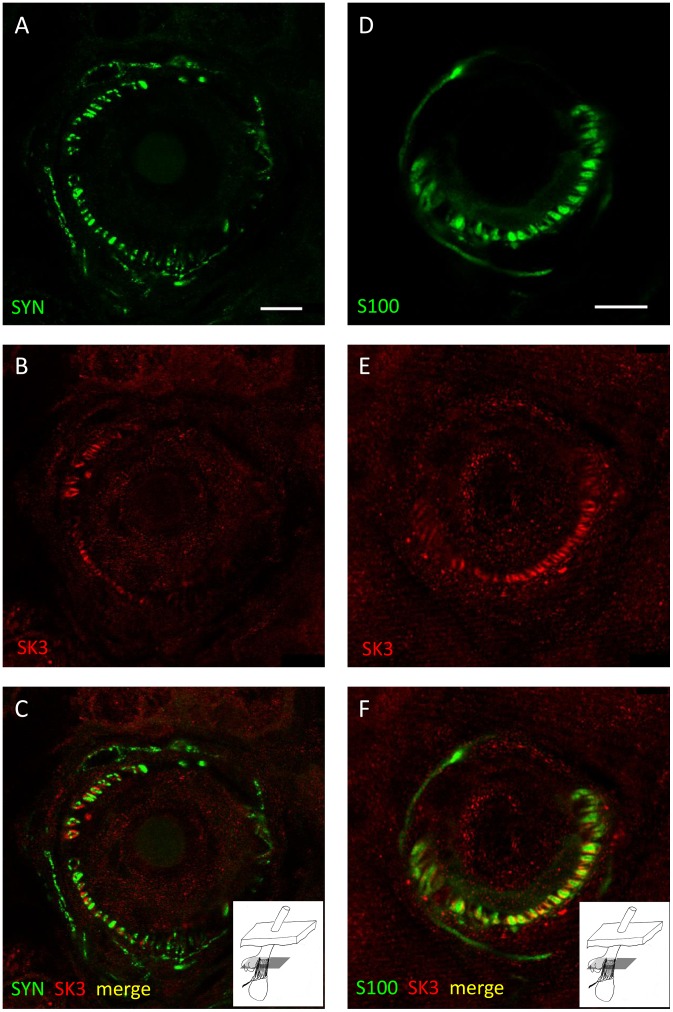
Evidence for the expression of SK3 in satellite glial cells of lanceolate endings. Lanceolate endings double labelled with anti-synaptophysin (SYN, green channel) and anti-SK3 (red channel) antibodies (A–C) and with anti-S100 (green channel) and anti-SK3 (red channel) antibodies (D–F). In each case the green (A, D) and red (B, E) channels are shown separately, as well as merged (C, F). Partial colocalisation of S100 with SK3 immunoreactivity, but only close association of SYN with SK3 antibodies, indicates that SK3 is present in satellite glial cells (SGCs), especially where they adjoin sensory terminals. Scale (in A, D) = 10 µm.

**Figure 7:**
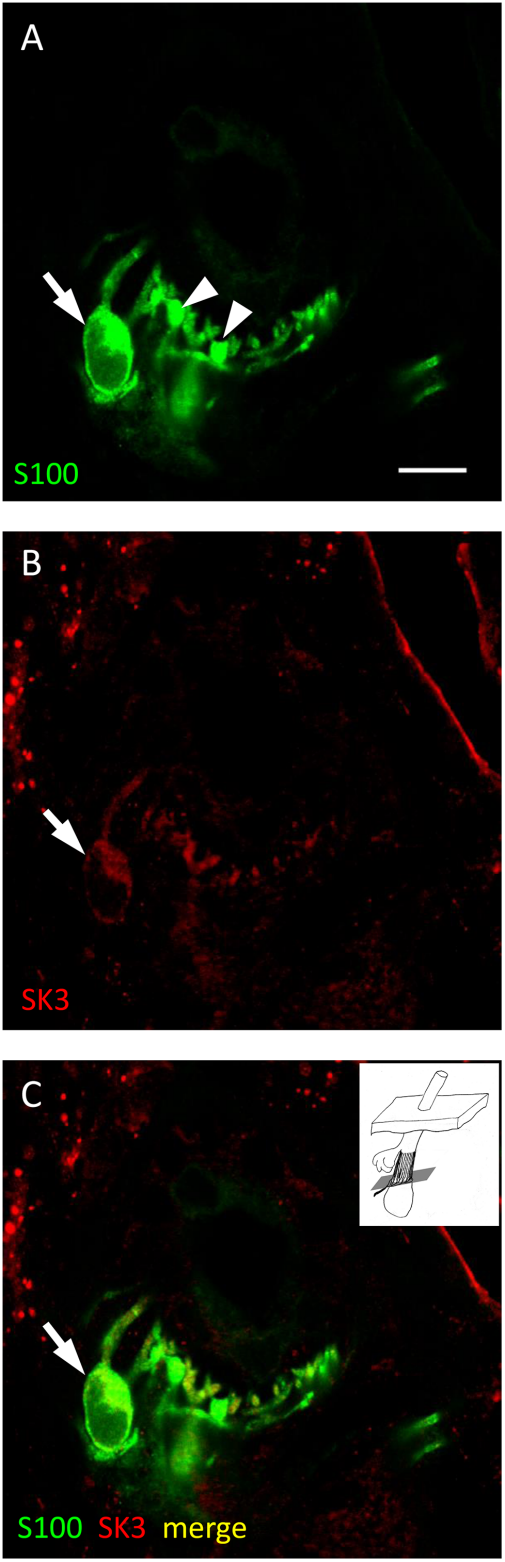
SK3 may be absent from some satellite glial cells in lanceolate endings. Lanceolate ending double labelled with anti-S100 (green channel) and anti-SK3 (red channel) antibodies shown separately and merged (A–C, respectively). SK3 immunoreactivity is clearly present in some satellite glial cells (SGC)s and their processes (arrows), whereas it is not apparent in others (arrowheads). Scale (in A) = 10 µm.

Although we found no evidence for SK3 in spindles, immunoreactivity to SK3 was found in restricted locations in lanceolate endings. Double labelling demonstrated that SK3 immunoreactivity occurred in association with both SYN and S100 immunoreactivities, but that it was only partiallycolocalised with either. Rather, it seemed to be localised at or near the interface between terminals (represented by SYN) and SGC processes (represented by S100) (Fig. 6 and Movie S5). Manders’ coefficients were: for SYN/SK3 (mean M_x_ = M_SK3/SYN_ = 0.33; mean M_y_ = M_SYN/SK3_ = 0.63, n = 11); and for S100/SK3 (mean M_x_ = M_SK3/S100_ = 0.42; mean M_y_ = M_S100/SK3_ = 0.88, n = 15). There was no significant difference in a statistical comparison of M_SK3/SYN_ and M_SK3/S100_, whereas M_SYN/SK3_ and M_S100/SK3_ differed highly significantly (Table 1). SK3 immunoreactivity was detectable in the cell bodies and processes of some SGCs; however, not all S100 immunoreactive profiles also showed SK3 reactivity, even when closely adjacent in the same section (Fig. 7 and Movie S6). These results are consistent with a restricted expression of SK3 in SGCs, especially where their processes are closely adjacent to sensory terminals. It is also possible that not all SGCs express SK3, and we cannot exclude the possibility, on the present evidence, of limited expression in the sensory terminals.

#### ASIC2 colocalisation pattern for comparison with SK channels

**Figure 8:**
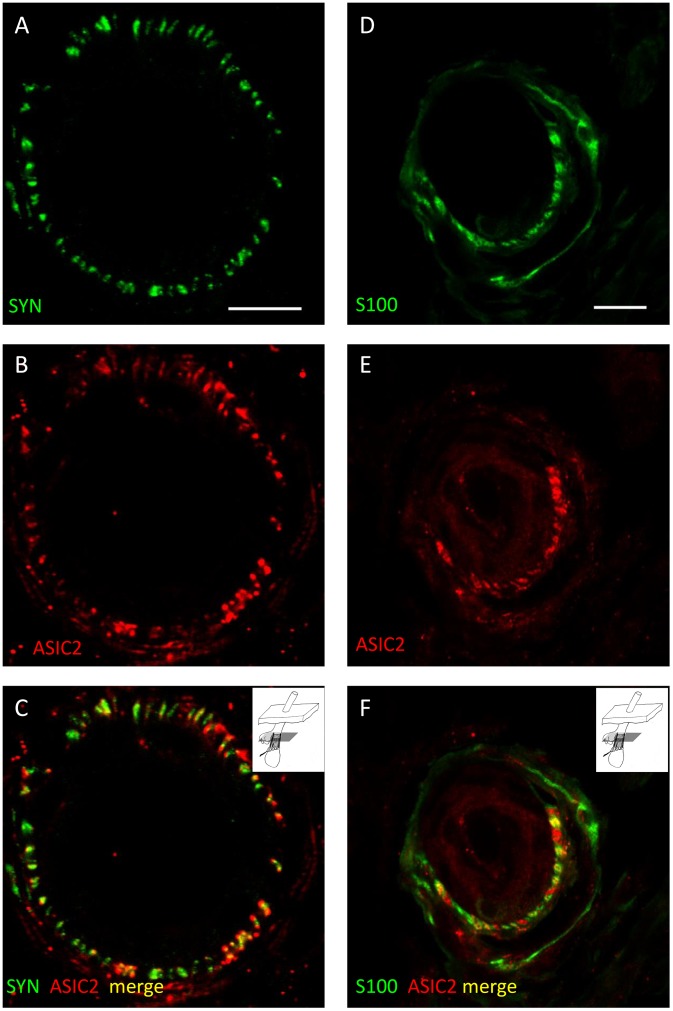
Partial colocalisation of ASIC2 and S100 reflects close association between sensory terminals and glial cells. Lanceolate endings double labelled with anti-synaptophysin (SYN, green channel) and anti-ASIC2 (red channel) antibodies (A–C) and with anti-S100 (green channel) and anti-ASIC2 (red channel) antibodies (D–F). In each case the green (A, D) and red (B, E) channels are shown separately, as well as merged (C, F). Since ASIC2 is not expressed in satellite glial cell (SGC) bodies, partial colocalisation, or close association, of both SYN and S100 with ASIC2 immunoreactivity indicates that ASIC2 is present in sensory terminals at, or close to their interface with SGCs. Scale (in A, D) = 10 µm.

ASIC2 immunoreactivity showed a similarly very restricted distribution in lanceolate endings to that of SK3, and also relatively low colocalisation with both SYN and S100 immunoreactivities (Fig. 8 and Movie S7). Manders’ coefficients were: for SYN/ASIC2 (mean M_x_ = M_ASIC2/SYN_ = 0.66; mean M_y_ = M_SYN/ASIC2_ = 0.67, n = 20); and for S100/ASIC2 (mean M_x_ = M_ASIC2/S100_ = 0.48; mean M_y_ = M_S100/ASIC2_ = 0.79, n = 15). Neither corresponding pair of coefficients differed significantly (M_x_ nor M_y_) (Table 1). In this case, however, there was no evidence of ASIC2 expression in SGC cell bodies or their processes, except where sensory terminals were also likely to be present. Conversely, there is clear evidence that ASIC2 (BNaC1α) is expressed in the sensory axons and terminals [12] and the restricted distribution in relation to SYN immunoreactivity in our own results is consistent with ASIC2 being localised especially at the sensory terminal membrane, in particular, the regions where it is known to be exposed. That is, at the edges of the long axis of the lanceolate terminal blade closest to, and furthest from, the hair shaft where there are gaps between the SGC processes [17].

### SK1

**Figure 9:**
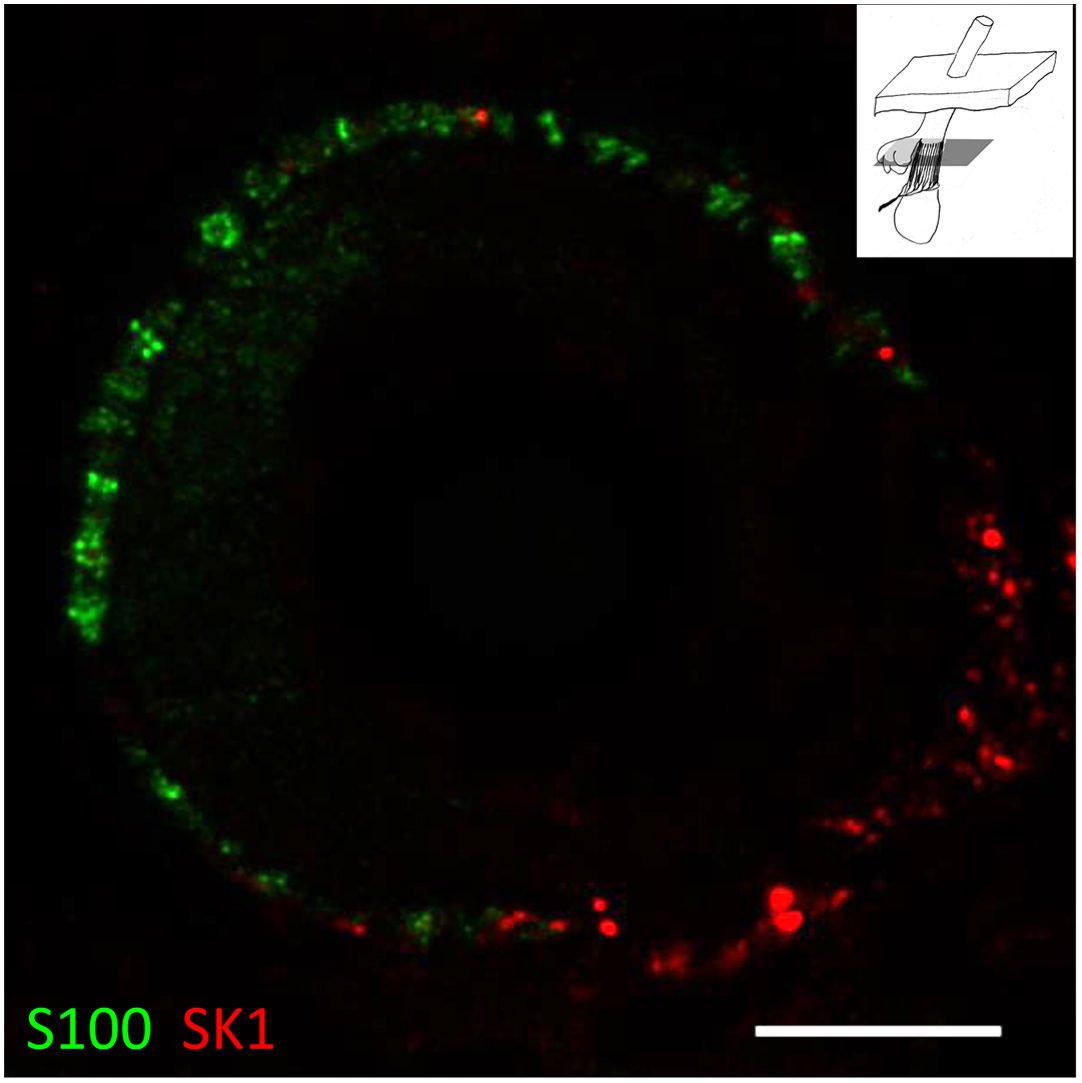
Example of SK1 expression in unidentified structure close to lanceolate ending. Lanceolate ending double labelled with anti-S100 (green channel) and anti-SK1 (red channel) antibodies. Immunoreactivity to S100 is present in processes of satellite glial cells (SGCs), and to SK1 in unidentified structures. Although the latter resemble axons it seems unlikely that is what they are, as there are no associated glial cell processes close to them. Scale = 10 µm.

We did not detect immunoreactivity to SK1 in the terminals either of sensory endings in muscle spindles or of lanceolate endings; however, there was some specific labelling with anti-SK1 antibody in unidentified axon-like structures, apparently located immediately beneath the lanceolate endings around some hair follicles (Fig. 9).

## Discussion

### Opening the black box – low-threshold sensory mechanotransduction in mammals

Despite their different locations, structural organisations and associations with very different accessory cells, the sensory endings of muscle spindles and the lanceolate endings of hair follicles share some fundamentally important properties in common, together with other cutaneous, joint and muscle afferents, and some visceral afferents such as baroreceptors [18]. Thus, all of these are low-threshold mechanoreceptors, responding to low force stimuli, often of minute amplitude; and all are formed of the peripheral sensory terminals of primary afferent axons whose cell bodies are located in the dorsal-root or cranial nerve ganglia. They also share in common the fact that the molecular basis of their mechanosensitivity is unknown. In some cases, such as the muscle spindle, classical neurophysiology has provided us with very detailed input:output (I/O) properties where the inputs are well-defined and precisely controlled mechanical stimuli, and the outputs are the resulting spike trains in the afferent axons (see, for example, [19]). In such experiments the overall process of mechanosensory transduction is treated as a black box, whose transfer function can, at least in principle, be determined from the I/O properties alone (see, for example, [20]). This treatment is very useful in bioengineering, but it is clearly unsatisfactory for our understanding of a fundamentally important biophysical process. For that we need to see inside the ‘black box’.

Much of our recent work has centred on the role of a glutamatergic system mediated by SLVs in mechanosensory terminals [6], [7]. The rate of SLV turnover is activity dependent, and experimental manipulation of the system alters the sensory ending’s I/O properties, at least of the muscle spindle, so it is feasible that the system is part of an automatic gain control of the mechanosensory black box, operating with a time course of seconds to minutes. We encountered the importance of Ca^2+^-activated K^+^ channels, both SK and BK, in the course of investigating the possible role of voltage-gated Ca^2+^ channels in SLV recycling in muscle spindles [9]. SLV recycling is a Ca^2+^-dependent process, so we were surprised initially to find that blocking P/Q Ca^2+^ channels enhanced rather than inhibited muscle-spindle sensory responses to stretch; however, as P/Q channels are frequently associated with K_Ca_ channels, we also tried blocking SK and BK channels and found that blocking either or both types produced similar effects to P/Q blockage [10]. It might be thought likely that these channels would be localised especially at sites in the sensory endings where action potentials are generated and where they could be particularly effective in regulating the firing rate. Heminodes, of which there may be several in any one sensory ending and which are located in preterminal branches, are thought to be the most important such sites, but they were not amenable to study in the present work where we have concentrated on the sensory terminals and associated accessory cells. We have carried out preliminary observations on the immunohistochemistry of BK, which indicate that it is not present in the sensory terminals of either muscle spindles or lanceolate endings (FCS,RWB), but we have yet to investigate whether immunoreactivity to BK or SK can be detected at heminodes.

### SK2 in sensory terminals

In the light of the previous consideration, our clear evidence for SK2 expression throughout the sensory terminals of both muscle spindles and lanceolate endings is all the more remarkable. In the very large terminals of muscle spindles much of the immunoreactivity was found within the terminals, though it was not completely colocalised with SYN, the SLV marker. Any SK2 localised internally is presumably sequestered in a reserve pool, as its functional site must surely be the sensory terminal membrane. The much smaller terminals of the lanceolate endings do not allow us to decide whether or not SK2 is similarly localised in part within the terminals as well as in their membranes, but the higher value of Manders’ coefficient M_x_ = M_SK2/SYN_ in lanceolate (0.90) as compared to muscle spindle endings (0.74) is at least consistent with this. Conversely, M_y_ = M_SYN/SK2_ is somewhat higher for muscle spindles (0.88) than for lanceolate endings (0.81), which might reflect the additional expression of SK2 in the SGCs that are a prominent feature of lanceolate endings, but are absent from muscle spindles.

The sensory terminals are, of course, thought to be the sites where receptor potentials are produced in response to the mechanical deformation of the terminals resulting in gating of stretch-activated channels in their membranes. Hunt et al. (1978) [4] succeeded in recording muscle-spindle receptor potentials from the parent axons, rather than the inaccessible terminals, by blocking action potentials with tetrodotoxin. They were able to attribute certain features of the receptor potential to K^+^ influx, but they also showed that removal of external Ca^2+^ had no effect on these features and presumably, therefore, the K^+^ currents responsible were not due to Ca^2+^-activated channels. This is a very important observation in the context of our results, as it suggests that the SK2 channels of the sensory terminals may require much greater, transient, depolarisations to invade the terminals by backfiring action potentials in order to activate P/Q channels associated with them, and that the role of the P/Q-SK2 channel complex within the terminals may therefore concern registration of the fact of action potential firing rather than modulation of the receptor potential itself. In contrast to the results of Hunt et al. (1978), Kruse and Poppele (1991) [5] found very clear evidence for a role for K_Ca_ channels in the mid-frequency dynamics of the muscle-spindle primary ending’s response to small-amplitude sinusoidal stretch, which they modelled as a negative feedback pathway within the process of receptor potential generation. What is perhaps particularly significant is that Kruse and Poppele (1991) had not blocked impulse activity and were, in fact, recording the responses as impulse activity from the parent afferent nerve fibre. Our finding of SK2 immunoreactivity in the sensory terminals may therefore be seen as providing further support to the idea of such a feedback pathway.

### SK2 and SK3 in satellite glial cells

In lanceolate endings spatial resolution with immunofluorescence was rarely sufficient to separate clearly the immunoreactivity of the sensory terminals and their associated SGC processes, even in single confocal planes. The quantitative data analysis must therefore be interpreted using additional qualitative information, provided by examination of SGC bodies and sensory axons in the region of the hair follicles deep to the palisade endings. At this level, at least, we could be sure that immunoreactivity to both SK2 and SK3 was present in SGCs, but in sensory axons only that to SK2 was present. Consistent with such a pattern of expression, at the level of the sensory terminals, we found Manders’ coefficients to be generally high for SK2 colocalisation with both SYN and S100 (all 4 values of M_x_ and M_y_ ranged between 0.81 and 0.96), but only M_y_ = M_S100/SK3_ was high (0.88) for SK3 colocalising with S100, implying that almost all the SK3 immunoreactivity colocalised with that of S100. The converse, however, was not true (M_x_ = M_SK3/S100_ = 0.42). Together with the low, quantitative, colocalisation of SK3 with SYN (M_x_ = M_SK3/SYN_ = 0.33, M_y_ = M_SYN/SK3_ = 0.63), this reflects the very restricted expression of SK3 apparently close to the interface between the sensory terminals and the SGC processes.

Individual sensory terminals of the lanceolate ending are very closely invested by processes of SGCs, and are otherwise exposed only on their inner and outer edges at narrow gaps between pairs of SGC processes. The SGCs are therefore well placed to influence the flow of any receptor current generated by the sensory terminals, in addition to perhaps playing a role in transmission of mechanical stimuli to the terminals. Our demonstration of the presence of SK2 and SK3 in SGCs, in particular their processes, suggests that one possible contribution they may make is the regulation of [K^+^] in the extraterminal space.

Our findings concerning the expression of SK3 in SGCs are consistent with previous reports of its presence in other types of glia, both centrally in astrocytes of the rat supraoptic nucleus [21], and peripherally in SGCs of the trigeminal ganglion [22]. Increases in intracellular [Ca^2+^] in other types of glia have been shown to regulate neuronal activity [23], but the signal for activation of SK channels in SGCs of the hair follicle are unknown. In lanceolate endings of rat vibrissae, however, Takahashi-Iwanaga et al. (2008) [24] have described a dual system of intercellular Ca^2+^ signalling in SGCs that could conceivably activate SK channels, in response to direct mechanical stimulation of the SGC processes themselves. Mechanical stimulation results in local elevation of intracellular [Ca^2+^], the signal then being propagated intercellularly by purine- and gap-junction-mediated pathways, effectively coupling the SGCs into a functional network [24].

## Supporting Information

Mpg-format movies created using Imaris software (Bitplane , Zurich, Swizerland) from z-stacks of confocal images.

Movie S1 **3D surface rendering of image stacks showing the distinct cellular localisations of anti-SYN and anti-S100 labelling.** The original *en face* raw anti-SYN image stack (green) of the hair follicle palisade is followed by addition of the anti-S100 (red) of the glial cell, then any colocalisation volume is rendered in yellow. This composite is then rotated to alternative viewing aspects. Note the absence of any colocalisation volume (yellow), indicating complete cellular segregation, with no synaptophysin labelling in the S100-positive glial cell, or *vice versa*.
(ZIP)

Movie S2 **3D surface rendering of image stacks showing the distinct cellular localisations of anti-SYN and anti-S100 labelling.** The original *en face* raw anti-SYN image stack (green) of the hair follicle palisade is followed by addition of the anti-S100 (red) of the glial cell, then any colocalisation volume is rendered in yellow. This composite is then rotated to alternative viewing aspects. Note the absence of any colocalisation volume (yellow), indicating complete cellular segregation, with no synaptophysin labelling in the S100-positive glial cell, or *vice versa*.
(ZIP)

Movie S3 **3D surface rendering of image stack showing the relationship between anti-SK2 and anti-SYN labelling.** The original *en face* raw SK2 image stack (red) of the almost complete circle of the hair follicle palisade is followed by addition of the lanceolate terminal (synaptophysin, green) and colocalisation volumes (yellow), which is then rotated to alternative viewing aspects. Note the extensive fragment of glial cell body (bottom centre) labelled for SK2, and the circumferential and longitudinal labelling (in the z-axis) in the rest of the follicle. The white framework indicates the total volume of the section that was imaged. Solid surface rendering is then applied to the SYN, SK2 then colocalisation volumes. The original image volume and the frame are then removed. Zooming in on the lanceolate terminals (green) shows they are predominantly enclosed by a larger (glial) volume labelled for SK2. However, transparency of the red and green channels allows the extensive labelling of SK2 within the terminals (yellow) to be seen. A lower power view then confirms the SK2 labelling is within the terminals, not just overlapping labels directly in line sight from a single view point, as the non-colocalised SK2 and SYN labels are progressively peeled away, leaving only the surface rendered colocalisation volume, before being reinstated.
(ZIP)

Movie S4 **3D surface rendering of image stack showing another example of colocalisation of anti-SK2 with anti-SYN labelling in lanceolate terminals.** As for movie S1, but in an oblique section deeper within the follicle that contains much more extensive glial cell, and less nerve terminal, material.
(ZIP)

Movie S5 **3D surface rendering of image stack showing anti-SK3 (red) labelling is very predominantly in the glial cell processes, and only colocalises (yellow) with the terminal anti-SYN (green) labelling at the interface between the lanceolate endings and their enclosing glial cell processes.** Note in this follicle, sectioned at a more superficial level of the skin, there are no cell bodies but the glial cell processes are clearly interpolated between the lanceolate terminals.
(ZIP)

Movie S6 **Confocal optical section stack close to the base of a lanceolate ending, double-labelled with anti-SK3 (red channel) and anti-S100 (green channel) antibodies.** Colocalisation of the immunoreactivities may be seen in some SGCs and their processes, but not others, and may be followed in the 3 dimensions of the image stack. Compare with the endings shown in Figure 7D–F and Figure 8, labelled in the same way. (Avi-format movie created using Windows Moviemaker from z-stack of confocal images.).
(ZIP)

Movie S7 **3D surface rendering of an image stack to contrast the relationship of anti-ASIC2 and anti-SYN labelling with that for the SK channels above.** Note the extensive ASIC2 labelling (red) in both longitudinal and circumferential elements. While there is substantial anti-ASIC2 labelling in the glial cell processes, it is also found extensively within most of the lanceolate terminals, colocalising with anti-SYN labeling.
(ZIP)
